# 0035. Mitochondrial uncoupling contributes to fever in sepsis

**DOI:** 10.1186/2197-425X-2-S1-O8

**Published:** 2014-09-26

**Authors:** E Greco, N Arulkumaran, A Dyson, M Singer

**Affiliations:** Bloomsbury Institute of Intensive Care Medicine, University College London, London, UK

## Introduction

The major sources of body heat production are muscle activity, chemical reactions involving ATP synthesis and usage, coupled (oxidative phosphorylation) and uncoupled (proton leak) mitochondrial respiration. The cause of fever associated with sepsis has not been elucidated, particularly in sedated, ventilated patients who neither perform much voluntary skeletal muscle activity nor shiver.

## Objectives

To determine whether the excess heat production in febrile septic rats is mediated by mitochondrial uncoupling, and related to an increase in global oxygen consumption (VO_2_).

## Methods

Awake, previously cannulated (tunneled carotid and jugular lines), male Wistar rats (approx. 300g body weight) were placed in metabolic cages to measure VO_2_. Core temperature was measured intermittently with a rectal probe. Twenty-four hours later, sepsis was induced by i.p. injection of faecal slurry. Sham animals received i.p. saline. Intravenous fluid resuscitation (10 ml/kg/h crystalloid) was started 2 h later. At 6 and 24 hours, animals were randomized to receive an infusion of either the mitochondrial uncoupler dinitrophenol (DNP) (30 mg/Kg) or n-saline over 1 hour. Wilcoxon Rank Sum test was used to compare groups and two-way ANOVA was used to compare change of continuous variables from baseline between groups, with p< 0.05 considered significant.

## Results

**Figure 1:**
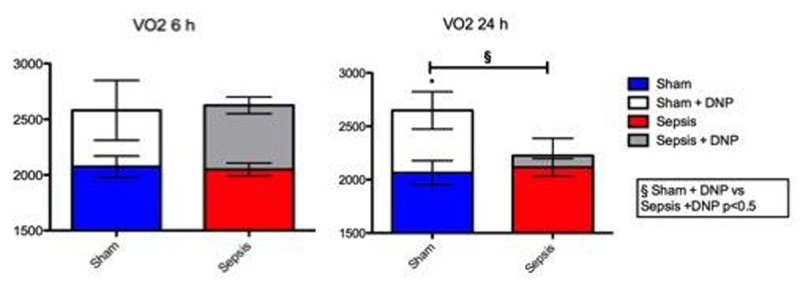
Figure 1

**Figure 2:**
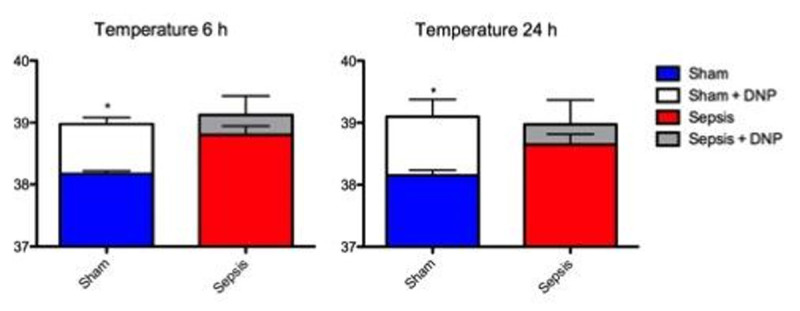
Figure 2

Sham animals were euthermic at 6 and 24h, and their VO_2_ was similar to baseline. Infusion of DNP produced similar rises in both temperature and VO_2_ (p< 0.05) at both timepoints (Figure 1). By contrast, the septic animals were febrile at 6h and 24h (p< 0.05 vs baseline) and DNP only induced a small, non-significant rise in temperature to the same level as seen in shams (Figure 2). Whereas 6h and 24h VO_2_ values were similar to shams, the effect of DNP on VO_2_ was similar to sham animals at 6h but significantly reduced at the 24h timepoint (Figure 1).

## Conclusions

In febrile septic animals, mitochondrial uncoupling with DNP only produced a small rise in temperature at 6h and 24h, and a subnormal VO_2_ response at 24h. This implies that increased mitochondrial uncoupling was already active in septic rats and this may explain their fever. The proportion of VO_2_ directed towards ATP-coupled respiration also appears to be reduced.

## References

[1] Rolfe DF, Brown GC (1997). Physiol Rev.

